# Glycopolymers stabilize protein folding and protein–protein interactions via enthalpic interactions

**DOI:** 10.1002/pro.70403

**Published:** 2025-12-22

**Authors:** Sabrina M. Richter, Neal Brook, Alex J. Guseman

**Affiliations:** ^1^ Department of Chemistry and Biochemistry University of California San Diego California USA

**Keywords:** 19F NMR, glycocalyx, macromolecular crowding, protein folding

## Abstract

Macromolecular crowding is ubiquitous in physiological environments, perturbing the thermodynamics and kinetics of proteins via excluded volume and nonspecific chemical interactions. While crowding has been well studied in vitro and in cells, the inert sugar polymers used to simulate crowding lack the chemical characteristics of biomolecules. Emerging studies guide the development of more relevant models of crowding in the cell, but little work has been done to discern crowding effects on proteins at the cell surface. Using ^19^F NMR, we measure how protein stability, folding, and intermolecular interactions are modulated by three glycopolymers abundant at the cellular exterior. Biologically relevant glycopolymers including heparin, hyaluronic acid, and mucin significantly stabilize the folding of the N‐terminal domain of the Drk‐SH3 protein. These interactions are enthalpically stabilizing, emphasizing the importance of chemical interactions for biologically relevant crowders. We further show that these glycopolymers stabilize a homodimer formed by the A34F variant of GB1, demonstrating that biological crowders not only affect isolated proteins, but also influence how proteins interact with one another. Crowding is more complex than simple ideas of volume exclusion suggest, and our work guides a more comprehensive understanding of protein crowding in the context of the glycocalyx, the last frontier of the cell.

## 1 INTRODUCTION

Biological concentrations of macromolecules range from 100 to 400 g/L (Zimmerman & Trach, 1991). In these crowded and heterogeneous environments, proteins are influenced by nonspecific interactions between macromolecules which are absent in dilute solutions (Minton, 1981; Sarkar et al., 2013). These interactions, including hardcore steric repulsions and chemical interactions, modulate different aspects of protein functionality, including the equilibrium thermodynamics and kinetics of protein folding (Christiansen et al., 2013; Gorensek‐Benitez et al., 2017; Miklos et al., 2010; Monteith & Pielak, 2014; Smith et al., 2016), protein diffusion (Cravens et al., 2015; Ye et al., 2013), rates of catalysis (Acosta et al., 2017; Rastogi & Chowdhury, 2021; Wilcox et al., 2021), and protein–protein interactions (Guseman, Perez Goncalves, et al., 2018a; Guseman & Pielak, 2017; Guseman, Speer, et al., 2018b; Heo et al., 2022). Hard‐core repulsions manifest themselves entropically and result from excluded volume effects as two molecules are unable to occupy the same space (Minton, 1981). Hardcore repulsions stabilize the most compact state of the protein, which often is the folded state. Chemical interactions can be attractive or repulsive, depending on the chemical features of the macromolecules involved; repulsive chemical interactions are stabilizing to protein folding and protein–protein interactions, while attractive interactions are destabilizing (Sarkar et al., 2013). These interactions have been well studied in vitro and in living cells with a focus on how proteins fold and interact with each other in the cellular interior (Chu et al., 2022; Gorensek‐Benitez et al., 2017; Guseman & Pielak, 2017; Smith et al., 2016; Stewart et al., 2023; Zhou, 2006). Here, we look to expand this understanding to the last frontier of the cell, the glycocalyx.

The cellular glycocalyx is a dense mesh of glycopolymers that coats the outside of the cell, where it plays a fundamental role in processes including cell motility, adhesion, viral and bacterial infection, and oncogenesis (Kanyo et al., 2020; Kuo et al., 2018; Möckl, 2020). These functions are potentiated through its chemical and physical characteristics. The chemical composition of the glycocalyx is hallmarked by an abundance of proteoglycans and glycoproteins (Reitsma et al., 2007). Proteoglycans include a protein core attached to one or several linear glycosaminoglycan (GAG) polymers spanning hundreds to thousands of kDa and richly functionalized with negatively charged chemical groups (Iozzo & Schaefer, 2015; Merry et al., 2022). Glycoproteins consist of larger protein cores, to which smaller and often branched carbohydrates are anchored (Kearns et al., 2024). Physically, the glycocalyx creates a barrier of entry to the cell membrane as it can extend tens of nanometers up to five micrometers beyond the cell surface (Kuo et al., 2018). The glycocalyx varies in density, glycan composition, and thickness from organism to organism, and is also influenced by factors such as cell type, metabolism, and cell cycle state (Kanyo et al., 2020; Kuo et al., 2018; Möckl, 2020; Reitsma et al., 2007). Proteins that reside in the glycocalyx often undergo conformational exchange between an inactive state and an active state, and this interchange is tightly regulated (Chataigner et al., 2020; Harpole & Delemotte, 2018).

The structural and chemical complexity of the glycocalyx renders it challenging to reconstitute, and studies which have aimed to simulate crowding effects at the cell surface have used the same synthetic sugar polymers routinely used to mimic crowding of the cellular interior (Chapanian et al., 2014; Lagrange et al., 2025). Here, we characterize the crowding effects of two GAG polymers, heparin and hyaluronic acid, and the highly glycosylated protein mucin, which together are representative of the heterogenous sugar polymers that occupy the exterior of the cell (Figure S1). Heparin is a 3–30 kDa polysaccharide comprised of repeating GlcUAα1‐4GlcNAc disaccharide units, notable for its high levels of sulfonation (Casu & Lindahl, 2001). It is one of the most abundant constituents of the glycocalyx (Reitsma et al., 2007). Hyaluronic acid is a linear, carboxylated GAG made of repeats of GlcUA*β*1‐3GlcNAc*β*1‐4, distinguished for its exceptional length upwards of 5 MDa (Merry et al., 2022). Mucins are a class of glycoproteins rich in serine and threonine residues that serve as the backbone for branched O‐linked glycans (Kearns et al., 2024). Here, we use ~15 kDa heparin, 50 kDa hyaluronic acid, and a heterogenous mixture of sialylated mucins isolated from porcine stomach.

Using two model systems, we compare the crowding effects of these charged glycopolymers relative to ficoll, a synthetic, uncharged sucrose polymer routinely used to generate crowded conditions. First, we measure how these crowders change the equilibrium thermodynamics and kinetics of protein folding, using the N‐terminal SH3 domain of the *Drosophila* adapter protein Drk (SH3) as a model. SH3 exists in dynamic equilibria between a folded and unfolded state, and exchange between these states can be efficiently quantified by ^19^F NMR (Bezsonova et al., 2005; Gorensek‐Benitez et al., 2017; Smith et al., 2016; Stadmiller & Pielak, 2018). To further characterize these physiological crowders, we measure their effects on the stability of a simple homodimer of the A34F variant of GB1 (Jee et al., 2008). GB1 is a thoroughly studied globular protein, and dimerization of the A34F mutant is highly sensitive to changes in environmental conditions (Chu et al., 2022; Guseman & Pielak, 2017; Guseman, Speer, et al., 2018b; Rydeen et al., 2018; Speer et al., 2021). The two‐state equilibria of both systems make them amenable for NMR‐based studies of protein dynamics.

## 2 RESULTS

### 2.1 Glycopolymers enthalpically stabilize the folded structure of SH3

**FIGURE 1:**
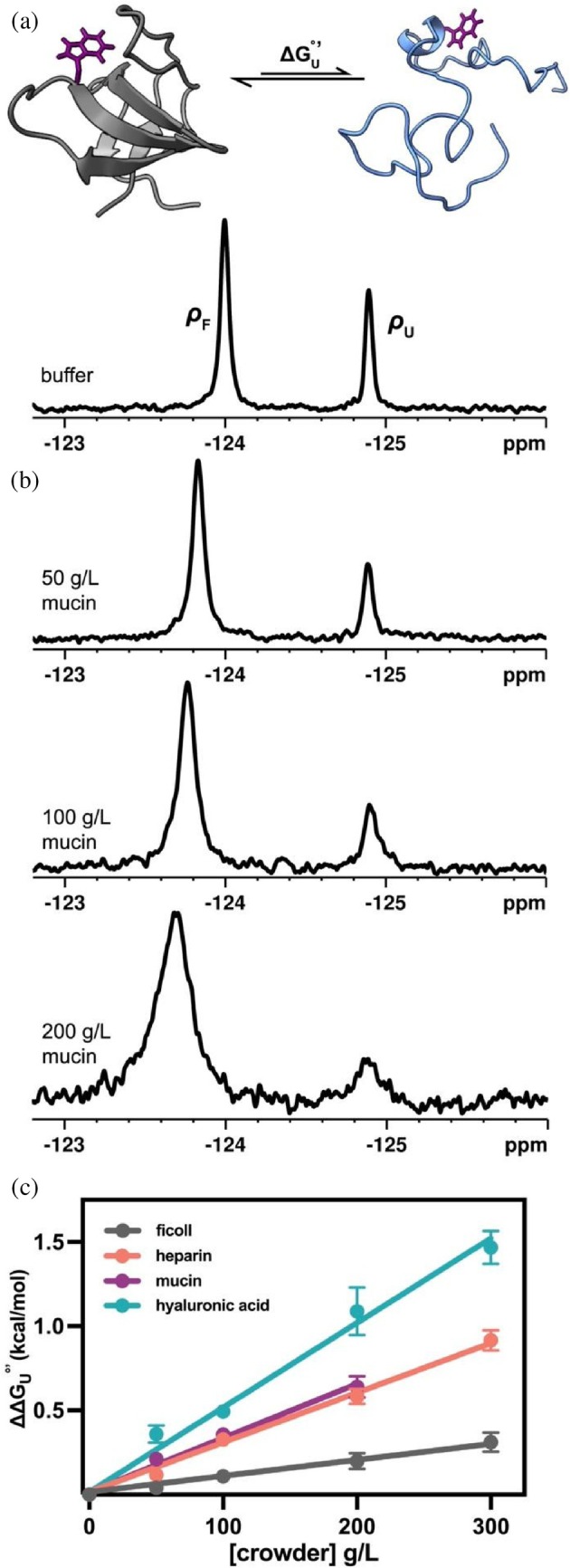
(a) top, structure of SH3 (PDBID2A36) in its folded (gray) and unfolded (blue) state, with fluorine‐labeled W36 colored purple; bottom, ^19^F NMR spectra of SH3 in pH 7.0 buffer at 298 K, with two resonances corresponding to folded (ρ_F_) and unfolded (ρ_U_) populations. (b) representative stacked ^19^F NMR spectra of SH3 in increasing concentrations of mucin in pH 7.0 buffer, 298 K. (c) comparison of change in SH3 modified standard state free energies of unfolding relative to buffer across increasing crowder concentrations.

SH3 has a standard state free energy of unfolding (${∆G}_{U}^{{}^{\circ}\prime }$) of ~0.5 kcal/mol (Smith et al., 2016). It coexists in a folded and unfolded state, and exchange between these states is slow on the NMR timescales (Zhang & Forman‐Kay, 1995). By fluorine‐labeling the sole tryptophan residue of SH3 with 5‐fluoroindole, populations of each state can simultaneously be quantified by ^19^F NMR (Figure 1a). Using this two‐state equilibrium, we measured how SH3 stability is perturbed by glycopolymers in concentrations as high as 300 g/L. Protein stability can be quantified by the modified standard free energy of unfolding (Equation 1),

(1) $$∆G_{U}^{{{}^{\circ}}^{\prime }}=-\text{R}\;\text{ln}\;\text{ln}\;\left(\frac{\rho_{U}}{\rho_{F}}\right),$$

where *R* is the ideal gas constant, *T* is the absolute temperature, and ρ_F_ and ρ_U_ are the populations of SH3 folded and unfolded, respectively. All the crowding conditions we generated stabilize the folded structure of SH3, and these effects are more prominent in physiological conditions compared with ficoll (Figures 1b, c and S2 and Table S1). At 300 g/L, ficoll stabilizes SH3 by 0.31 ± 0.08 kcal/mol. At the same concentration, heparin and hyaluronic acid stabilize SH3 by 0.92 ± 0.08 kcal/mol and 1.5 ± 0.1 kcal/mol, respectively. Mucin also stabilizes protein folding, but concentrations above 200 g/L could not be measured due to its extremely high viscosity. All three glycopolymers bias SH3 almost entirely to its folded state (Figures 1b and S2).

The resonances of SH3 broaden in crowded environments, and resonance broadening scales with crowder concentration (Figures S2 and S3 and Table S2). Nonspecific chemical interactions between proteins and macromolecules can manifest themselves via peak broadening, slower tumbling times and faster relaxation dynamics (Barbieri et al., 2015; Smith et al., 2016). To compare relaxation dynamics in different crowding environments, we estimated transverse relaxation times from linewidths (Mladenov & Dimitrov, 2001). Estimated T2 times are ~2–4 fold faster in 300 g/L crowder conditions compared with buffer, and these effects are more outstanding in biological crowders compared to ficoll. Thus, we suspected that stabilization of SH3 by biological crowders arises from chemical interactions.

**FIGURE 2:**
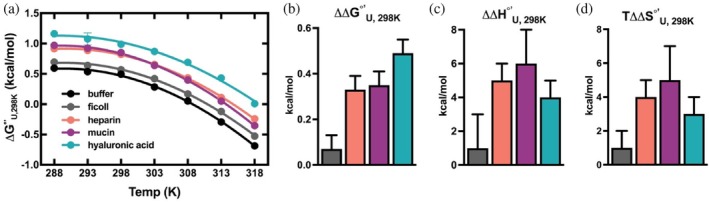
(a) Temperature curves of SH3 in buffer or 100 g/L of each crowder. Data are shown as the mean ± SD of three measurements. Some error bars are not visible where the error is smaller than the data points. (b–d) Comparison of the changes in standard state free energy (${∆∆G}_{U}^{{}^{\circ}\prime }$), enthalpy (${∆∆H}_{U}^{{}^{\circ}\prime }$), and entropy (${T∆∆S}_{U}^{{}^{\circ}\prime }$) of unfolding in each crowder condition compared to buffer.

To extract the entropic and enthalpic contributions of each crowding condition, we then measured the temperature dependence of ${∆G}_{U}^{{}^{\circ}\prime }$. Data were fit to the integrated Gibbs‐Helmholtz equation (Equation 2) as described by Smith and colleagues (Smith et al., 2016),

(2) $$∆G_{U,T}^{{{}^{\circ}}^{\prime }}=∆H_{U,T_{\mathrm{ref}}}^{{{}^{\circ}}^{\prime }}-T∆S_{U,T_{\mathrm{ref}}}^{{{}^{\circ}}^{\prime }}+∆C_{p,U}^{{{}^{\circ}}^{\prime }}\left(T-T_{\mathrm{ref}}-\text{T}\;\text{ln}\;\text{ln}\frac{T}{T_{\mathrm{ref}}}\right),$$

where *T*_ref_ refers to the melting temperature (when ⍴_U_ = ⍴_F_). At 100 g/L, ficoll marginally stabilizes SH3 by 0.07 ± 0.06 kcal/mol, but both the entropic and enthalpic effects of ficoll are indistinguishable from buffer (Figure 2 and Table S3). Contrary to the idea of volume exclusion, we found that heparin, hyaluronic acid, and mucin entropically de*stabilize* SH3. Entropic destabilization of SH3 has previously been reported in other sugar polymers (Olgenblum et al., 2025; Smith et al., 2016; Stewart et al., 2023), and it is possible that the expected steric penalty of unfolding is not prevalent in crowder concentrations of 100 g/L used here. Enthalpically, the folded state of SH3 is favored in all three glycopolymers, and these effects are of greater magnitude than the entropic penalty of folding, leading to net stabilization. Chemical interactions thus have the dominant effect on protein stability.

### 2.2 Glycopolymers have disparate effects on protein folding kinetics

**FIGURE 3:**
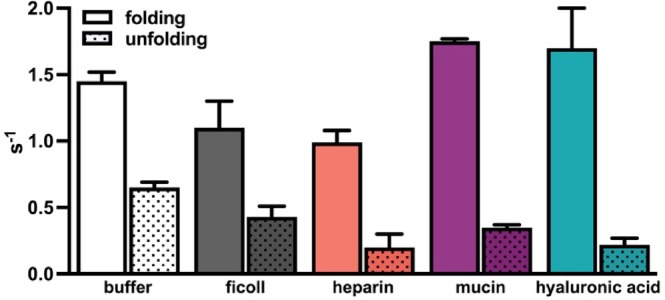
Folding (*k*_f_) and unfolding (*k*_u_) rates of SH3 in 100 g/L each crowder condition, calculated from 2D ^19^F EXSY experiments. Folding is shown as solid bars and unfolding as dotted bars.

Using 2D ^19^F EXSY experiments, we measured the folding (*k*_f_) and unfolding (*k*_u_) rates of SH3. Data were analyzed according to Farrow et al. (1994; Gustafson et al., 2017). In buffer, the folding rate of SH3 is 1.45 ± 0.07 s^−1^ and the unfolding rate is 0.65 ± 0.04 s^−1^ (Figures 3 and S4 and Table S4). The uncharged sugar polymer ficoll slows both processes with a folding rate of 1.1 ± 0.2 s^−1^ and an unfolding rate of 0.43 ± 0.08 s^−1^. Extending this analysis to the glycopolymers, we found that heparin also slows both folding (0.99 ± 0.09 s^−1^) and unfolding (0.2 ± 0.1 s^−1^). For mucins and hyaluronic acid, the rate of folding increases to 1.75 ± 0.02 s^−1^ and 1.7 ± 0.3 s^−1^, respectively, while the rate of unfolding decreases to 0.35 ± 0.02 s^−1^ and 0.22 ± 0.05 s^−1^, respectively.

### 2.3 Biologically relevant glycopolymers stabilize protein–protein interactions

**FIGURE 4:**
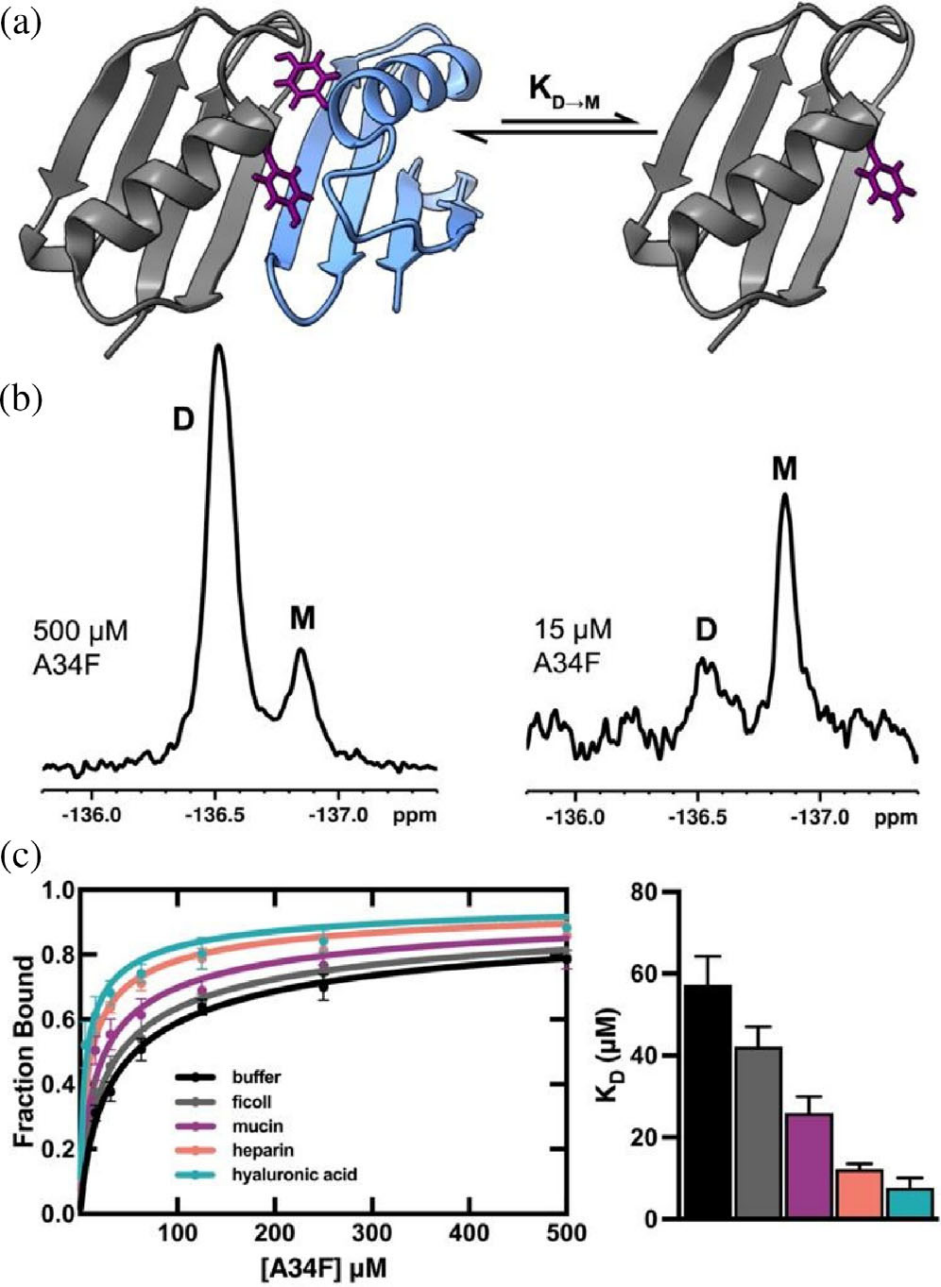
(a) GB1 A34F (PDBID2RMM) dimer and monomer. 3‐fluoro‐labeled Y33 residues are shown in purple. (b) ^19^F NMR spectra of A34F with two Y33 resonances for the dimer (D) and monomer (M). Full spectra are shown in Figure S5. Populations of each state change in a concentration‐dependent manner. Spectra were taken in pH 7.0 buffer, 298 K. (c) binding curves from ^19^F NMR titrations of A34F in different crowding conditions (left) and comparison of dissociation constants in each condition (right). Data is shown as the mean ± SD of three measurements.

Finally, we investigated how these biologically relevant glycopolymers bias protein–protein interactions using the T2Q A34F variant of GB1 (A34F), which exists in equilibria between a monomer and dimer (Jee et al., 2008). Y33 resides at the dimerization interface and experiences different chemical environments as a monomer and dimer; exchange between these states is slow, and fluorine‐labeling the tyrosine residues of A34F yields two resonances for Y33 by ^19^F NMR, one corresponding to each state (Figure 4a, b). We measured the stability of the A34F dimer in each crowding environment by using ^19^F NMR titrations at several protein concentrations (P_T_) between 15 and 500 μM. Peak integrations were used to determine the fraction monomer (*f*_M_) and dimer (*f*_D_) at each concentration, and dissociation constants (*K*_D⟶M_) were determined by fitting data to Equation (3) in Prism using nonlinear least squares fit.

(3) $$f_{D}=\frac{\left[4P_{T}+K_{D⟶M}-\sqrt{K_{D⟶M}^{2}+8P_{T}K_{D}}\right]}{4P_{T}}$$

Binding energies could then be determined using Equation (4):

(4) $$∆G_{D\to M}^{{{}^{\circ}}^{\prime }}=-\text{R}\;\text{ln}\;\text{ln}\;K_{D⟶M},$$

where *R* is the ideal gas constant and *T* is the absolute temperature.

In buffer, we measured a *K*_D→M_ of 57 ± 6 μM, in excellent agreement with previous reports (Table 1 and Figure 4c) (Guseman & Pielak, 2017). Stabilization of the dimer is minimal in ficoll (*K*_D→M_ = 42 ± 5 μM). The binding affinity of the dimer is enhanced by ~2 fold in 100 g/L mucin and ~4 fold in 100 g/L heparin. In hyaluronic acid, the *K*_D→M_ is 8 ± 2 μM and the dimer is stabilized by 1.2 ± 0.2 kcal/mol relative to buffer.

**TABLE 1:** Thermodynamic parameters for dimerization of A34F.

	K_D→M_ (μM)	${∆G}_{D⟶M}^{{}^{\circ}\prime }$ (kcal/mol)	${∆∆G}_{D⟶M}^{{}^{\circ}\prime }$ (kcal/mol)
Buffer	57 ± 6	4.4 ± 0.1	–
Ficoll	42 ± 5	4.6 ± 0.1	0.2 ± 0.1
Mucin	26 ± 4	4.9 ± 0.1	0.5 ± 0.1
Heparin	12 ± 1	5.4 ± 0.1	1.0 ± 0.1
Hyaluronic acid	8 ± 2	5.6 ± 0.2	1.2 ± 0.2

## 3 DISCUSSION

Historically, discussions of macromolecular crowding were limited to hard‐core repulsions, where proteins were treated as inert spheres inherently biased towards compact structures in crowded environments (Minton, 1981). As protein stability was later measured in concentrated protein environments, cell lysates, and in living cells, classic crowding theories could not explain the enthalpic interactions between a test protein and its environment. Thus, modern crowding theory has evolved to understand that crowding includes an entropic packing component arising from volume exclusion, and an enthalpic chemical component arising from the electrostatic properties of a crowder and protein. Recent studies reflect the limited efficacy of synthetic sugar polymers as mimetics of the cellular interior, as these polymers lack charged functional groups (Chu et al., 2022; Danielsson et al., 2015; Miklos et al., 2011; Sarkar et al., 2014; Smith et al., 2016). Inside cells, chemical interactions between protein surfaces have a greater influence on protein stability and interactions than excluded volume effects, and synthetic sugar polymers such as ficoll and dextran have been poor models of macromolecular crowding in the cytoplasm (Smith et al., 2016). Our work expands upon this paradigm, but using the component sugar polymers of the glycocalyx as crowders, which contain charged functional groups such as sulfates and carboxylic acids. We demonstrated how three physiological glycopolymers influence protein thermodynamics and kinetics, where chemical interactions were the dominant force. The uncharged polymer ficoll failed to mimic the crowding effect from charged physiological glycopolymers, reflecting a need for more comprehensive models of macromolecular crowding that account for the chemical features of biological macromolecules.

The surfaces of SH3 and A34F are negatively charged at physiological pH, rendering the folding (SH3) and dimerization (A34F) of these proteins highly sensitive to charge–charge interactions (Figure S6). Both serve as useful models of how proteins may behave in the glycocalyx, with approximately half of human extracellular proteins weakly acidic (pI 4–7) (Kurotani et al., 2019). We hypothesized that the negatively charged glycopolymers would enthalpically stabilize SH3 folding. By measuring the temperature dependence of the folding free energy, we were able to calculate the enthalpy (${∆H}_{U}^{{}^{\circ}\prime }$) and entropy (${T∆S}_{U}^{{}^{\circ}\prime }$) for each condition, where the enthalpic stabilization outweighed the entropic destabilization. We observed an interaction enthalpy (${∆∆H}_{U}^{{}^{\circ}\prime }$) for SH3 folding of 4–6 kcal/mol for these physiologically relevant glycopolymers compared to uncharged ficoll, whose interaction enthalpy was not distinguishable from dilute solution. Interestingly, we noted that hyaluronic acid was more stabilizing to SH3 than heparin. While both polymers have charged functional groups, hyaluronic acid is decorated with negatively charged carboxylate groups, while heparin is decorated with sulfates and was expected to have a greater negative charge and thus an expected greater effect. We observed that heparin had a greater ${∆∆H}_{U}^{{}^{\circ}\prime }$ (5 ± 1) than the hyaluronic acid ${∆∆H}_{U}^{{}^{\circ}\prime }$ (4 ± 1), but heparin also demonstrated a greater entropic destabilization than hyaluronic acid. As a result, hyaluronic acid demonstrated a larger ${∆∆G}_{U}^{{}^{\circ}\prime }$. The differences of these effects may be explained by the size difference of the polymers, as the hyaluronic acid used in this study was 50 kDa and the heparin was 15 kDa. The interplay between polymer size, shape, and crowding effect is different between polymers and in different model systems, thus we are hesitant to overinterpret these data (Olgenblum et al., 2025; Stewart et al., 2023; Zosel et al., 2020). We found that glycosylated mucins also stabilized SH3. The mucins used in this study were heterogenous as they were purified from porcine stomach. Although they were less chemically defined than the GAGs, mucins are heavily sialylated and carry negative charge via the carboxylate functional groups present on sialic acid, similar to how GAGs convey negative surface charge through negatively charged chemical groups. These polymers generated stabilizing chemical interactions for SH3 as observed by the largest ${∆∆H}_{U}^{{}^{\circ}\prime }$ of the glycopolymers (6 ± 2), but mucin also had the largest entropic penalty to folding. These results demonstrate the potential for glycosylated proteins to generate stabilizing chemical environments for proteins and have effects comparable to negatively charged GAG polymers.

The mechanisms of SH3 stabilization by chemical interactions have been extensively studied in the context of protein crowders. *Smith* et al. demonstrated that crowders such as BSA and lysozyme have preferential interactions with the unfolded state of the protein as observed by changes in linewidths, a proxy for transverse relaxation rate T_2_, and by changes in the kinetic parameters of folding *k*_*u*_ and *k*_*f*_ (Smith et al., 2016). Extending this analysis to our study, each glycopolymer we studied displayed a concentration‐dependent increase in linewidths for both the folded and unfolded states; however, the folded states show ~3× more broadening than the unfolded states, indicative of stabilizing interactions with the folded state (Table S2 and Figure S2). Using ^19^F‐EXSY we measured the rates of folding and unfolding. We observed that hyaluronic acid and mucin increased the folding rate and decreased the unfolding rate, suggestive of interactions with the folded state (Table S2 and Figure S3). For heparin, these interactions are less clear; we saw a decrease in both the folding and unfolding rates, suggesting a balance between interactions with both states. Different from previously studied protein crowders that interact with the unfolded state of SH3, the biologically relevant glycopolymers we used stabilized SH3 folding via interactions with the folded state.

Extending these studies to protein–protein interactions, we used the model homodimer GB1 A34F to investigate how glycopolymer crowding affects protein dimerization. In physiological crowding environments, the A34F dimer was stabilized by 0.5–1.2 kcal/mol and binding of the dimer increased 2–6 fold. Following the same trends as protein folding, all three glycopolymers stabilized the dimer more than ficoll, and hyaluronic acid had the most significant effects. While we are unable to measure the interaction enthalpy of A34F, previous studies by Pielak and colleagues have demonstrated that A34F follows similar trends in chemical interactions to SH3, where charge–charge repulsions are stabilizing (Guseman & Pielak, 2017; Guseman, Speer, et al., 2018b; Speer et al., 2021), suggesting that the stability of the dimer is also enthalpically motivated in this case. Importantly, this shows that the glycopolymers which compose the glycocalyx influence protein–protein interactions in the same fashion as protein folding.

The glycocalyx is a dynamic network of glycopolymers that is constantly changing as cells experience different stimuli (Biering et al., 2021; Möckl, 2020; Rehm et al., 2007). The data presented in this study demonstrate a potential mechanism in which the glycocalyx can regulate biological processes, as remodeling of the glycocalyx in response to stimuli or stress may alter the conformational landscapes of proteins native to the glycocalyx. Thus, if an extracellular domain of a signaling protein has a signaling inactive state A and a signaling active state B that undergoes conformational change to drive a signaling process, under homeostatic conditions a tightly regulated thermodynamic barrier exists. As the glycocalyx gets remodeled, these barriers may shift, modulating the energetic differences between state A and B and either increasing or decreasing the thermodynamic barrier for these transitions. This presents an exciting avenue for further investigation.

## 4 MATERIALS AND METHODS

### 4.1 Protein expression and purification

A pET‐3a plasmid encoding the sequence for SH3 was transformed into BL21(DE3) *E. coli* cells and plated overnight on LB agar supplemented with 100 μg/mL carbenicillin. The next day, a single colony was used to inoculate 60 mL LB broth supplemented with the appropriate antibiotic. The culture was grown for 14 h at 37°C. The next day, the overnight culture was split into 3 × 1 L flasks of M9 minimal media supplemented with 100 μg/mL carbenicillin. Cells were grown at 37°C, 200 rpm to an OD of 0.6, at which point 60 mg 5‐fluoroindole was added to each flask. Cultures continued to shake for 30 min, then were cooled to 18°C and protein expression was induced with isopropyl β‐d‐1‐thiogalactopyranoside (IPTG) to a final concentration of 0.5 mM. Cultures were shaken at 18°C, 200 rpm for 16 h, then centrifuged at 4000×*g* for 20 min, and dry cell pellets were stored at −80°C.

Protein purification was performed as previously described (Stadmiller et al., 2017). Cell pellets were resuspended in lysis buffer (20 mM TRIS pH 8.5), lysed by sonication, and clarified by centrifugation at 24,000×*g* for 30 min. The soluble material was loaded on a HiTrap Q HP column on an ÄKTA go™ system, washed in lysis buffer, and eluted in the same buffer with a gradient of 0–1 M NaCl. Fractions containing SH3 were pooled, concentrated to 1 mL, and further purified by size exclusion chromatography on a Hiload Superdex 75 16/600 column. Pure protein fractions were dialyzed into 18.2 MΩ cm^−1^ water for 12 h and concentrated to <2 mLs at 4°C, 4000×*g* in a 5 kDa cutoff concentrator. Final protein purity was determined by SDS‐PAGE and ESI‐MS (expected mass 6878 Da, observed 6879 Da), and concentration was determined by nanodrop (8480 M^−1^ cm^−1^) (Figure S7). Samples were aliquoted for NMR, lyophilized, and stored at −80°C. A pET‐3a plasmid containing the sequence for the T2Q A34F variant of GB1 (here referred to as A34F) was transformed into BL21 (DE3) *E. coli* cells. Expression of GB1 followed the procedure described above with two exceptions. A34F was grown in ^15^N enriched M9 media, and 60 mg 3‐fluorotyrosine was added to cell cultures at an OD of 0.6 instead of 5‐fluorindole. A34F was purified as previously described (Guseman & Pielak, 2017) following the same procedure described in the paragraph above. Protein concentration was determined by nanodrop (9970 M^−1^ cm^−1^) and purity was confirmed by SDS‐PAGE and ESI‐MS (expected mass 6393 Da, observed 6417 Da) (Figure S8).

## 5 NMR

Lyophilized samples of fluorine‐labeled SH3 were resuspended in 20 mM phosphate pH 7.0 in 10% D_2_O to a final concentration of 200 μM, with the appropriate glycopolymer. Heparin sodium (15 kDa), ficoll (70 kDa), and mucin were purchased from Sigma. Hyaluronic acid (50 kDa) was purchased from Fisher. Experiments were performed on a Bruker Ascend Evo spectrometer operating at a ^19^F Larmor frequency of 565 MHz equipped with a cryogenic TCI probe tunable to fluorine. 1D spectra of SH3 were acquired with an acquisition of 4096 points and a relaxation delay of 2 s, with an offset of −120 ppm and a sweep width of 30 ppm. The number of scans was either 128 or 256. For variable temperature experiments, spectra were collected at 288, 293, 298, 303, 308, 313, and 318 K. Spectra were acquired at 298 K before and after the temperature gradient to ensure reversibility of folding. For folding experiments, SH3 was suspended in the same buffer to a final concentration of 400 μM. A Bruker library NOESY experiment (*t*_mix_ = 20 ms, 75 ms (×3), 125, 175, 250, 400, 600, and 800 ms) was used to measure folding and unfolding rates. 2048 points were acquired in the f2 dimension with 128 points in the f1 dimension, across a sweep width of 10 ppm. 16 transients were acquired per increment, and the relaxation delay was 2 s. Titrations with GB1 were done using the same instrument. Protein was resuspended in 20 mM phosphate, pH 7.0 in 10% D_2_O to a concentration of 500 μM and serially diluted to 250, 125, 62.5, 31.2, and 15.6 μM in the same condition. 1D ^19^F spectra were acquired with an acquisition of 4096 points and a relaxation delay of 2 s, with an offset of −130 ppm and a sweepwidth of 30 ppm. The number of scans varied from 128 to 2048, depending on the protein concentration and crowder conditions.

Data were processed on Topspin 4.5.0. An exponential line broadening function of 10 Hz was applied to each FID before Fourier transformation. For 1D experiments with SH3, peak integrations were used to determine ⍴_U_ and ⍴_F_. For exchange experiments, the ratio of peak volumes was extracted, and the rates of exchange were calculated as described by Gustafson et al. (Gustafson et al., 2017). One mixing time was measured three times and the sample standard deviation was used to drive a Monte Carlo analysis (*n* = 1000) to propagate error to the rest of the data set (Smith et al., 2016). For 1D experiments with GB1, Y33 peak integrations were used to determine *f*_M_ and *f*_D_. Additional details of data fitting are described in the results section.

## AUTHOR CONTRIBUTIONS

**Sabrina M. Richter:** Investigation; writing – original draft; writing – review and editing; validation; formal analysis; conceptualization. **Neal Brook:** Investigation; writing – review and editing; data curation. **Alex J. Guseman:** Conceptualization; investigation; funding acquisition; writing – original draft; writing – review and editing; formal analysis; project administration; supervision; resources; validation.

## Supporting information


**FIGURE S1.** Chemical structures of two GAG polymers, heparin (left) and hyaluronic acid (right). While the identity of *R* and *R*′ for heparin is variable, there must be at least one SO_3_^−^ per repeating GAG disaccharide.
**TABLE S1.** Changes in thermodynamic stability of SH3 in crowding environments.
**FIGURE S2.** Representative ^19^F NMR spectra of 5‐fluorotryptophan‐labeled SH3 in ficoll, heparin, and hyaluronic acid. The upfield resonance corresponds to the unfolded population and the downfield resonance to the folded population. Assignments are as previously published (Evanics et al. 2006). All spectra were collected at pH 7.0, 298 K.
**FIGURE S3.** Linewidths of folded and unfolded state peaks in each condition at increasing crowder concentrations. Unfolded peak linewidths could not be determined above 200 g/L because the folded state is strongly biased. Data are shown as the mean ± SD of three measurements.
**TABLE S2.** Linewidths of SH3 folded and unfolded resonances and estimated T2 values (Mladenov & Dimitrov, 2001) in each condition.
**TABLE S3.** Thermodynamic parameters of SH3 at 298 K.
**FIGURE S4.** Representative EXSY curves for SH3 folding and unfolding in each glycopolymer at 100 g/L and fit to the EXSY model established by Farrow et al. (1994) Each curve represents the change in intensity over time for the fold state (blue), unfolded state (purple), and cross peaks (gold).
**TABLE S4.** Folding and unfolding rates of SH3 at 298 K.
**FIGURE S5.** Representative ^19^F NMR titration of GB1. Spectra of 3‐fluorotyrosine labeled GB1 show six peaks in total, two for each tyrosine. The rotamers of Y3 and Y45 are in slow exchange, and each show two resonances. Y33 shows two resonances, and peak ratios change with protein concentration. The upfield resonance corresponds to the monomer (M) and the downfield resonance to the dimer (D). Assignments are as previously published (Ye et al., 2013). All spectra were taken at pH 7.0, 298 K.
**FIGURE S6.** Electrostatic surface maps for SH3 (PDB ID2A36), and GB1 (A34F) (PDB ID2RMM), with positively charged surfaces shown in blue, negatively charged surfaces in red, and uncharged surfaces in white. Theoretical pI values were calculated from amino acid sequence using ProtCalc (Gasteiger et al. 2005).
**FIGURE S7.** Raw (left) and deconvoluted (right) ESI‐MS of SH3 labeled with 5‐fluoro‐tyrptophan. Peaks indicate protein with 0 (6861 Da) and 1 (6879 Da) fluorinated residue.
**FIGURE S8.** Raw (top) and deconvoluted (bottom) ESI‐MS of ^15^N‐enriched GB1 A34F labeled with 3‐fluorotyrosine. Peaks indicate protein with 0 (6364 Da), 1 (6382 Da), 2 (6399 Da), and 3 (6417 Da) fluorinated tyrosine residues.

## Data Availability

The data that support the findings of this study are available from the corresponding author upon reasonable request.
