# Supplementary material for: Glycopolymers stabilize protein folding and protein–protein interactions via enthalpic interactions
Source: Protein Sci. 2025 Dec 22;35(1):e70403. doi: 10.1002/pro.70403 (PMC12720779; doi:10.1002/pro.70403)
Supplement: Supplementary file 1 — FIGURE S1. Chemical structures of two GAG polymers, heparin (left) and hyaluronic acid (right). While the identity of R and R′ for heparin is variable, there must be at least one SO3 − per repeating GAG disaccharide. TABLE S1. Changes in thermodynamic stability of SH3 in crowding environments. FIGURE S2. Representative 19F NMR spectra of 5‐fluorotryptophan‐labeled SH3 in ficoll, heparin, and hyaluronic acid. The upfield resonance corresponds to the unfolded population and the downfield resonance to the folded population. Assignments are as previously published (Evanics et al. 2006). All spectra were collected at pH 7.0, 298 K. FIGURE S3. Linewidths of folded and unfolded state peaks in each condition at increasing crowder concentrations. Unfolded peak linewidths could not be determined above 200 g/L because the folded state is strongly biased. Data are shown as the mean ± SD of three measurements. TABLE S2. Linewidths of SH3 folded and unfolded resonances and estimated T2 values (Mladenov & Dimitrov, 2001) in each condition. TABLE S3. Thermodynamic parameters of SH3 at 298 K. FIGURE S4. Representative EXSY curves for SH3 folding and unfolding in each glycopolymer at 100 g/L and fit to the EXSY model established by Farrow et al. (1994) Each curve represents the change in intensity over time for the fold state (blue), unfolded state (purple), and cross peaks (gold). TABLE S4. Folding and unfolding rates of SH3 at 298 K. FIGURE S5. Representative 19F NMR titration of GB1. Spectra of 3‐fluorotyrosine labeled GB1 show six peaks in total, two for each tyrosine. The rotamers of Y3 and Y45 are in slow exchange, and each show two resonances. Y33 shows two resonances, and peak ratios change with protein concentration. The upfield resonance corresponds to the monomer (M) and the downfield resonance to the dimer (D). Assignments are as previously published (Ye et al., 2013). All spectra were taken at pH 7.0, 298 K. FIGURE S6. Electrostatic surface maps for SH3 (PDB ID2A36), and [file PRO-35-e70403-s001.docx]

**Supplementary Information for:**

**Glycopolymers stabilize protein folding and protein-protein interactions via enthalpic interactions**

**Sabrina M. Richter^1^, Neal Brook^1^, and Alex J. Guseman^1^***

**^1^Department of Chemistry and Biochemistry University of California San Diego, La Jolla California**

***Corresponding Author**

**
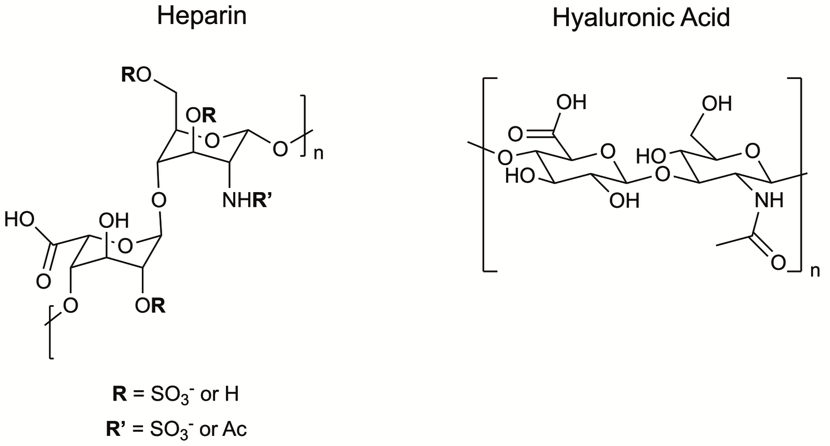
**

**Figure S1.** Chemical structures of two GAG polymers, heparin (left) and hyaluronic acid (right). While the identity of R and R’ for heparin is variable, there must be at least one SO_3_^-^ per repeating GAG disaccharide.

**Table S1.** Changes in thermodynamic stability of SH3 in crowding environments.

|  | $\boldsymbol{\Delta G}_{\boldsymbol{U}}^{\boldsymbol{^{\circ}'}}$ **(kcal/mol)** | $\boldsymbol{\Delta\Delta G}_{\boldsymbol{U}}^{\boldsymbol{^{\circ}'}}$ **(kcal/mol)** |
| --- | --- | --- |
| **buffer** | 0.50 ± 0.06 | - |
| **ficoll (g/L)** |  |  |
| 50 | 0.54 ± 0.01 | 0.04 ± 0.06 |
| 100 | 0.60 ± 0.02 | 0.11 ± 0.06 |
| 200 | 0.69 ± 0.05 | 0.20 ± 0.07 |
| 300 | 0.81 ± 0.06 | 0.31 ± 0.08 |
| **heparin (g/L)** |  |  |
| 50 | 0.61 ± 0.03 | 0.12 ± 0.06 |
| 100 | 0.82 ± 0.01 | 0.33 ± 0.06 |
| 200 | 1.08 ± 0.04 | 0.58 ± 0.07 |
| 300 | 1.41 ± 0.06 | 0.92 ± 0.08 |
| **hyaluronic acid (g/L)** |  |  |
| 50 | 0.86 ± 0.05 | 0.36 ± 0.08 |
| 100 | 0.99 ± 0.01 | 0.49 ± 0.06 |
| 200 | 1.6 ± 0.1 | 1.1 ± 0.2 |
| 300 | 2.0 ± 0.1 | 1.5 ± 0.1 |
| **mucin (g/L)** |  |  |
| 50 | 0.70 ± 0.01 | 0.21 ± 0.06 |
| 100 | 0.85 ± 0.01 | 0.36 ± 0.06 |
| 200 | 1.13 ± 0.06 | 0.64 ± 0.08 |


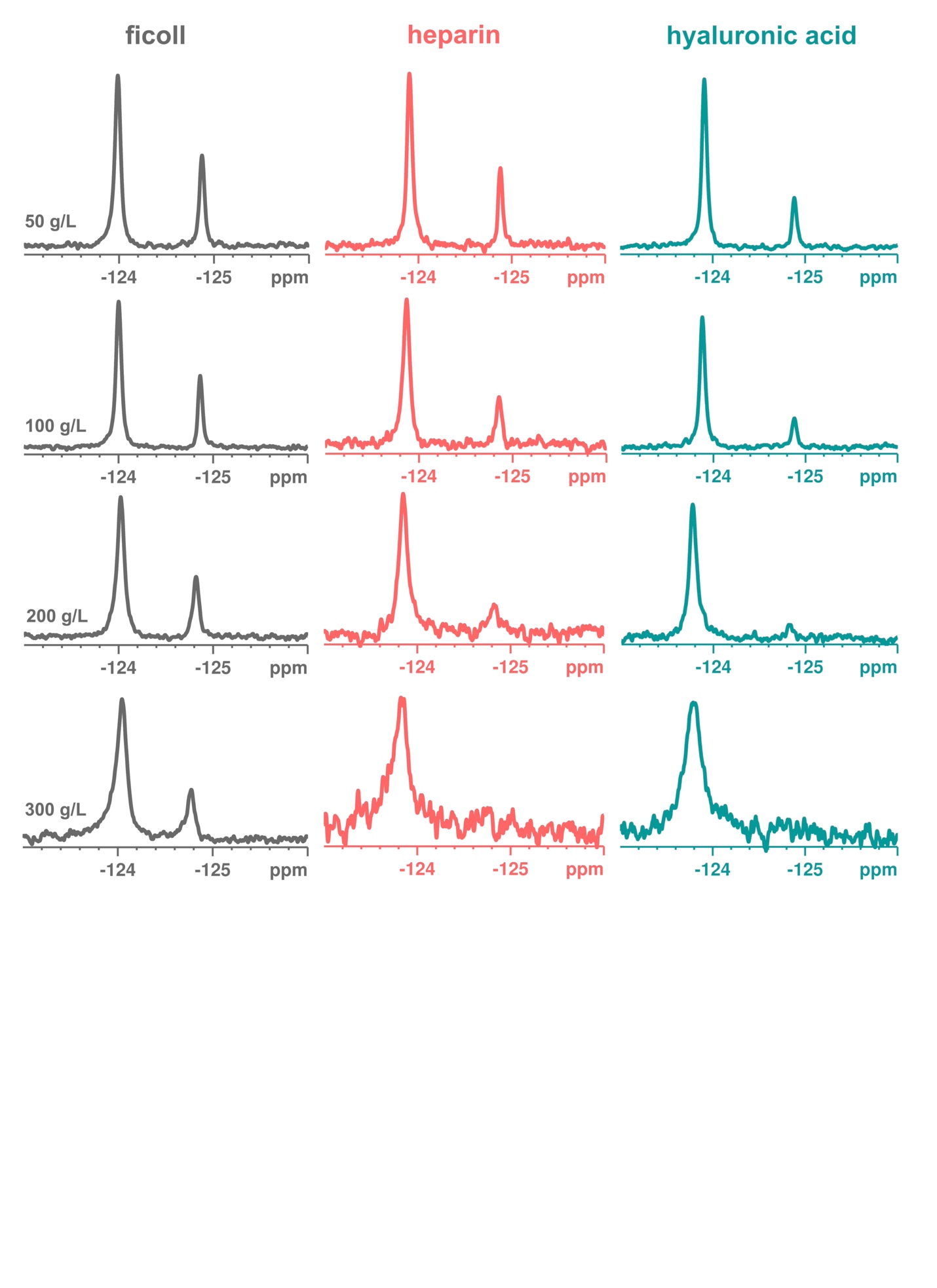
**Figure S2.** Representative ^19^F NMR spectra of 5-fluorotryptophan-labelled SH3 in ficoll, heparin, and hyaluronic acid. The upfield resonance corresponds to the unfolded population and the downfield resonance to the folded population. Assignments are as previously published.^1^ All spectra were collected at pH 7.0, 298K.


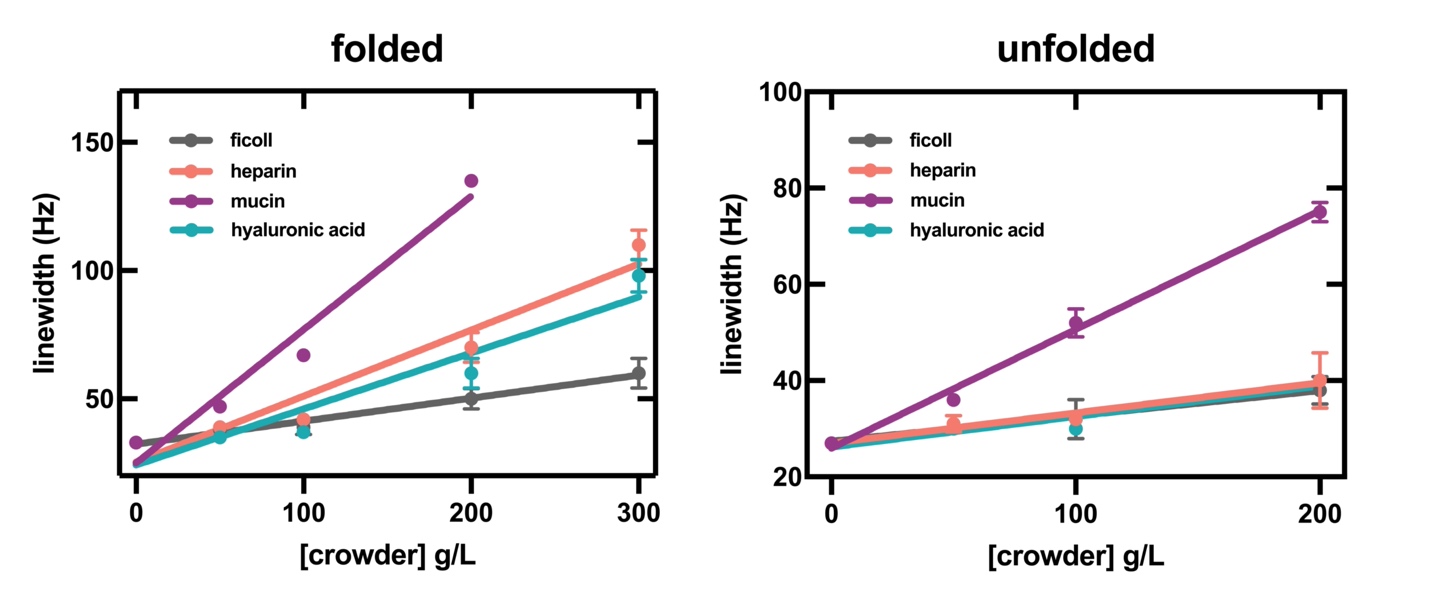


**Figure S3.** Linewidths of folded and unfolded state peaks in each condition at increasing crowder concentrations. Unfolded peak linewidths could not be determined above 200 g/L because the folded state is strongly biased. Data are shown as the mean ± SD of three measurements.

**Table S2.** Linewidths of SH3 folded and unfolded resonances and estimated T2 values^2^ in each condition.

|  |  | **linewidth (Hz)** | |  | **T2 (ms)** | |
| --- | --- | --- | --- | --- | --- | --- |
|  |  | **folded** | **unfolded** |  | **folded** | **unfolded** |
| **buffer** |  | 33 ± 1 | 27 ± 1 |  | 9.6 ± 0.3 | 11.8 ± 0.4 |
| **ficoll (g/L)** |  |  |  |  |  |  |
| 50 |  | 38 ± 1 | 31 ± 1 |  | 8.4 ± 0.2 | 10.3 ± 0.3 |
| 100 |  | 39 ± 5 | 32 ± 7 |  | 8 ± 1 | 10 ± 2 |
| 200 |  | 50 ± 7 | 38 ± 5 |  | 6.4 ± 0.9 | 8 ± 1 |
| 300 |  | 60 ± 10 | 40 ± 6 |  | 5.3 ± 0.9 | 8 ± 1 |
| **heparin (g/L)** |  |  |  |  |  |  |
| 50 |  | 39 ± 2 | 31 ± 3 |  | 8.2 ± 0.4 | 10 ± 1 |
| 100 |  | 42 ± 2 | 32 ± 1 |  | 7.6 ± 0.4 | 10.0 ± 0.3 |
| 200 |  | 70 ± 10 | 40 ± 10 |  | 4.6 ± 0.6 | 8 ± 2 |
| 300 |  | 110 ± 10 | n.d. |  | 2.9 ± 0.3 | n.d. |
| **hyaluronic acid (g/L)** |  |  |  |  |  |  |
| 50 |  | 35 ± 1 | 28 ± 1 |  | 9.1 ± 0.3 | 11.4 ± 0.4 |
| 100 |  | 37 ± 1 | 30 ± 2 |  | 8.6 ± 0.2 | 10.6 ± 0.7 |
| 200 |  | 60 ± 10 | 40 ± 10 |  | 5.3 ± 0.9 | 8 ± 2 |
| 300 |  | 98 ± 9 | NA |  | 3.2 ± 0.3 | n.d. |
| **mucin (g/L)** |  |  |  |  |  |  |
| 50 |  | 47 ± 1 | 36 ± 2 |  | 6.8 ± 0.1 | 8.8 ± 0.5 |
| 100 |  | 67 ± 1 | 52 ± 5 |  | 4.8 ± 0.1 | 6.1 ± 0.6 |
| 200 |  | 135 ± 3 | 100 ± 30 |  | 2.4 ± 0.1 | 3 ± 1 |

**Table S3.** Thermodynamic parameters of SH3 at 298K.

|  | $\Delta G_{U, 298K}^{{^{\circ}}^{'}}$  **(kcal/mol)** | $\Delta H_{U, 298K}^{{^{\circ}}^{'}}$  **(kcal/mol)** | $T\Delta S_{U, 298K}^{{^{\circ}}^{'}}$  **(kcal/mol)** | $\Delta C_{p, U, 298K}^{{^{\circ}}^{'}}$  **(kcal/mol)** |
| --- | --- | --- | --- | --- |
| **buffer** | 0.50 ± 0.06 | 18 ± 1 | 18 ± 1 | 0.93 ± 0.07 |
| **100g/L ficoll** | 0.57 ± 0.01 | 19.9 ± 0.9 | 19.1 ± 0.9 | 0.86 ± 0.05 |
| **100g/L heparin** | 0.82 ± 0.01 | 23.7 ± 0.3 | 22.2 ± 0.4 | 0.85 ± 0.06 |
| **100g/L hyaluronic acid** | 0.99 ± 0.01 | 22.5 ± 0.6 | 21.2 ± 0.5 | 0.8 ± 0.1 |
| **100g/L mucin** | 0.85 ± 0.01 | 24 ± 1 | 23 ± 1 | 0.93 ± 0.07 |
|  |  |  |  |  |
|  | $\Delta\Delta G_{U, 298K}^{{^{\circ}}^{'}}$  **(kcal/mol)** | $\Delta\Delta H_{U, 298K}^{{^{\circ}}^{'}}$  **(kcal/mol)** | $T\Delta\Delta S_{U, 298K}^{{^{\circ}}^{'}}$  **(kcal/mol)** | $\Delta\Delta C_{p, U, 298K}^{{^{\circ}}^{'}}$  **(kcal/mol)** |
| **100g/L ficoll** | 0.07 ± 0.06 | 1 ± 2 | 1 ± 1 | -0.08 ± 0.08 |
| **100g/L heparin** | 0.33 ± 0.06 | 5 ± 1 | 4 ± 1 | -0.09 ± 0.09 |
| **100g/L hyaluronic acid** | 0.49 ± 0.06 | 4 ± 1 | 3 ± 1 | -0.1 ± 0.1 |
| **100g/L mucin** | 0.35 ± 0.06 | 6 ± 2 | 5 ± 2 | 0.00 ± 0.09 |

**Figure S4**. Representative EXSY curves for SH3 folding and unfolding in each glycopolymer at 100 g/L and fit to the EXSY model established by Farrow et al.^3^ Each curve represents the change in intensity over time for the fold state (blue), unfolded state (purple), and cross peaks (gold).

**Table S4.** Folding and unfolding rates of SH3 at 298K.

|  | ***k*_f_ (s^-1^)** | ***k*_u_ (s^-1^)** |
| --- | --- | --- |
| **buffer** | 1.45 ± 0.07 | 0.65 ± 0.04 |
| **100g/L ficoll** | 1.1 ± 0.2 | 0.43 ± 0.08 |
| **100g/L heparin** | 0.99± 0.09 | 0.2 ± 0.1 |
| **100g/L mucin** | 1.75 ± 0.02 | 0.35 ± 0.02 |
| **100g/L hyaluronic acid** | 1.7± 0.3 | 0.22 ± 0.05 |


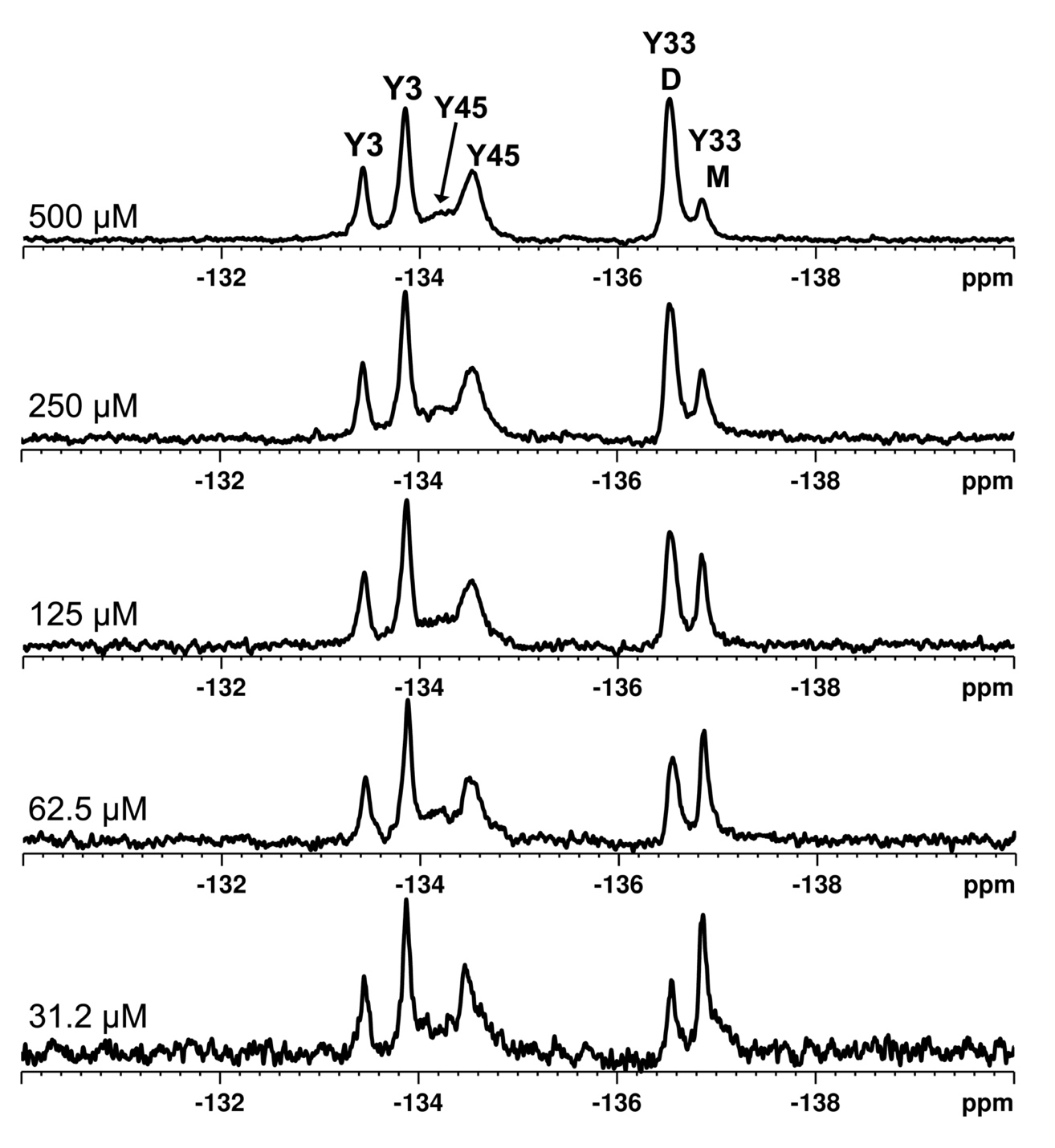


**Figure S5.** Representative ^19^F NMR titration of GB1. Spectra of 3-fluorotyrosine labelled GB1 show six peaks in total, two for each tyrosine. The rotamers of Y3 and Y45 are in slow exchange, and each show two resonances. Y33 shows two resonances, and peak ratios change with protein concentration. The upfield resonance corresponds to the monomer (M) and the downfield resonance to the dimer (D). Assignments are as previously published.^4^ All spectra were taken at pH 7.0, 298K.


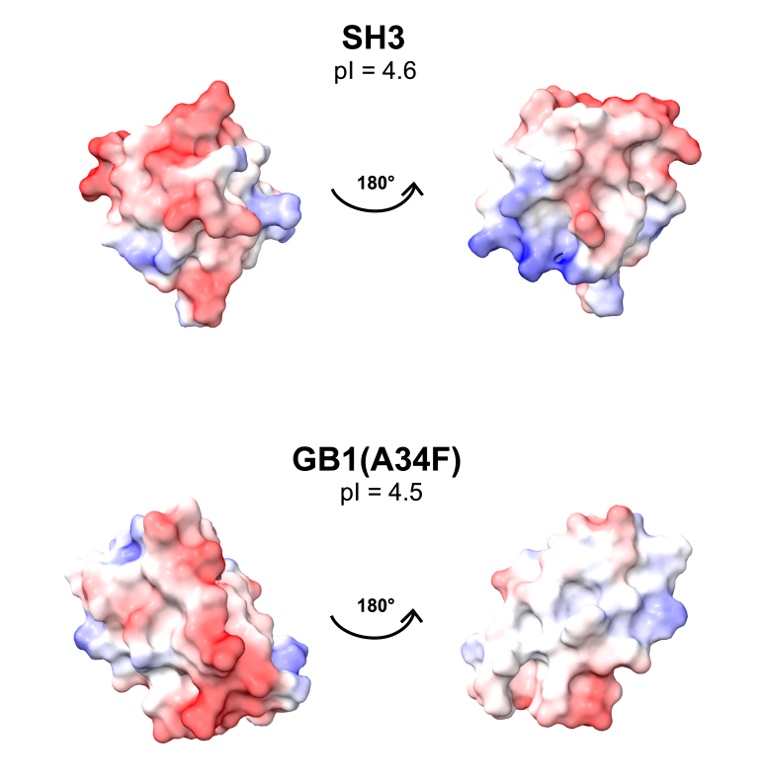


**Figure S6.** Electrostatic surface maps for SH3 (PDB ID 2A36), and GB1(A34F) (PDB ID 2RMM), with positively charged surfaces shown in blue, negatively charged surfaces in red, and uncharged surfaces in white. Theoretical pI values were calculated from amino acid sequence using ProtCalc.^5^


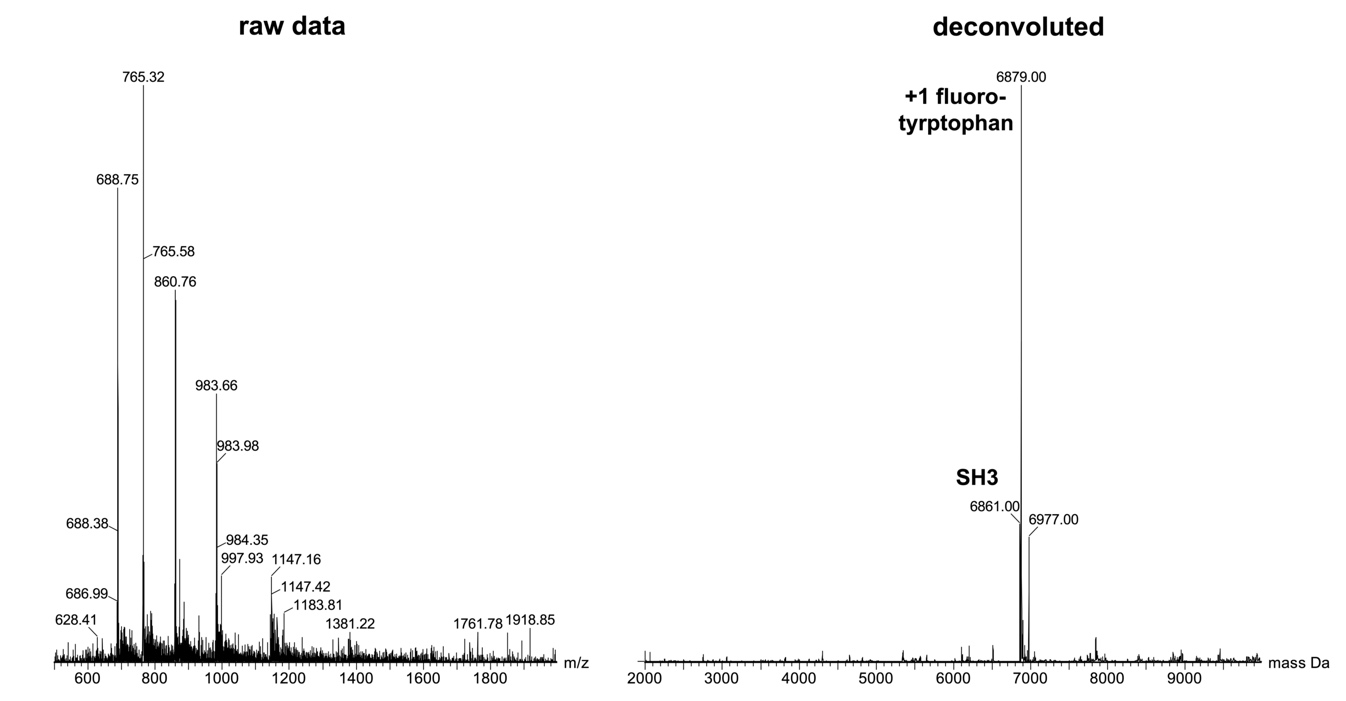


**Figure S7.** Raw (left) and deconvoluted (right) ESI-MS of SH3 labelled with 5-fluoro-tyrptophan. Peaks indicate protein with 0 (6861 Da) and 1 (6879 Da) fluorinated residue.


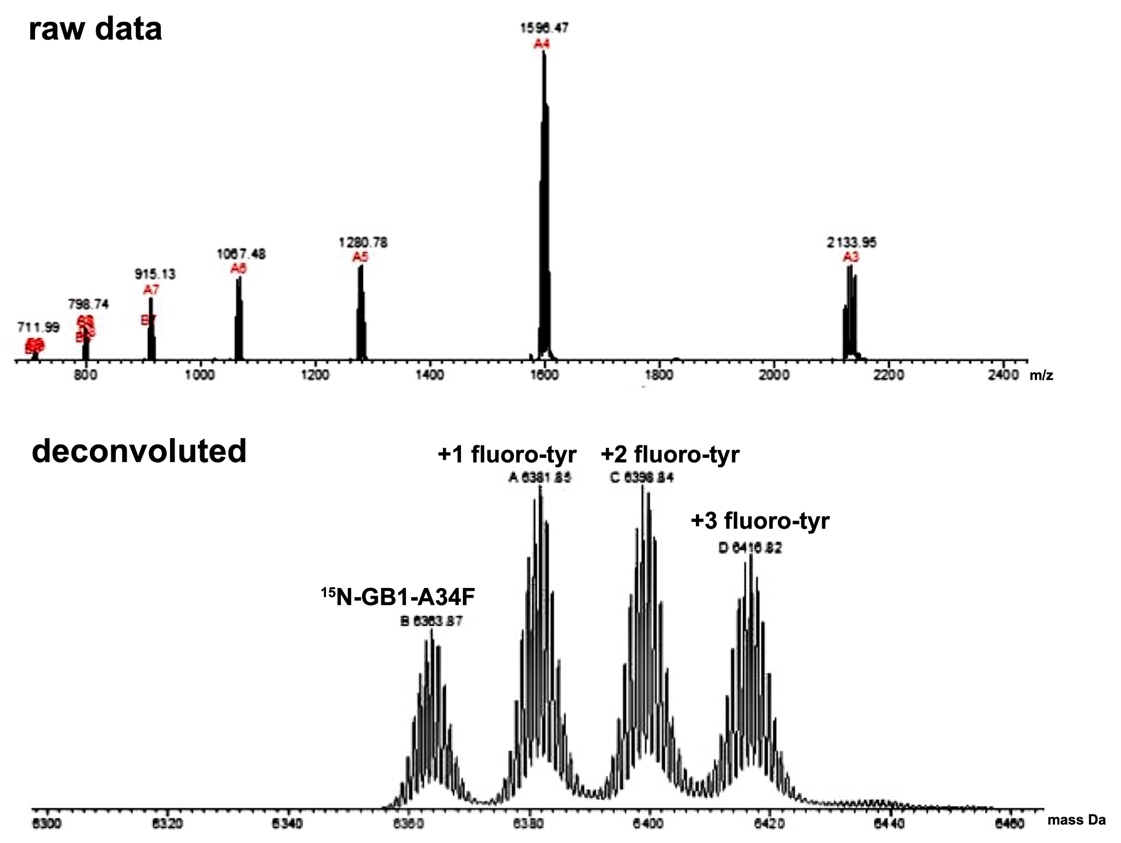


**Figure S8.** Raw (top) and deconvoluted (bottom) ESI-MS of ^15^N-enriched GB1 A34F labelled with 3-fluorotyrosine. Peaks indicate protein with 0 (6364 Da), 1 (6382 Da), 2 (6399 Da), and 3 (6417 Da) fluorinated tyrosine residues.

1. Evanics, F.; Bezsonova, I.; Marsh, J.; Kitevski, J. L.; Forman-Kay, J. D.; Prosser, R. S. Tryptophan Solvent Exposure in Folded and Unfolded States of an SH3 Domain by^19^ F and^1^ H NMR. *Biochemistry* **2006**, *45* (47), 14120–14128.
2. Mladenov, G.; Dimitrov, V. S. Extraction of *T*_2_ from NMR Linewidths in Simple Spin Systems by Use of Reference Deconvolution. *Magnetic Reson in Chemistry* **2001**, *39* (11), 672–680.
3. Farrow, N. A.; Zhang, O.; Forman-Kay, J. D.; Kay, L. E. A Heteronuclear Correlation Experiment for Simultaneous Determination of 15N Longitudinal Decay and Chemical Exchange Rates of Systems in Slow Equilibrium. *J Biomol NMR* **1994**, *4* (5), 727–734.
4. Ye, Y.; Liu, X.; Zhang, Z.; Wu, Q.; Jiang, B.; Jiang, L.; Zhang, X.; Liu, M.; Pielak, G. J.; Li, C. ^19^ F NMR Spectroscopy as a Probe of Cytoplasmic Viscosity and Weak Protein Interactions in Living Cells. *Chemistry A European J* **2013**, *19* (38), 12705–12710.
5. Gasteiger, E.; Hoogland, C.; Gattiker, A.; Duvaud, S.; Wilkins, M.R.; Appel, R.D.; Bairock, A. Protein Identification and Analysis Tools on the ExPASy Server. In *The Proteomics Protocols Handbook*; Walker, J.M., Ed.; Human Press, 2005, pp 571-607.
